# Association study between mannose-binding lectin haplotypes and X gene mutation of hepatitis B virus from treatment naïve patients

**DOI:** 10.18632/aging.101097

**Published:** 2016-11-07

**Authors:** Chenghao Su, Yong Lin, Qianguo Mao, Daitze Wu, Lina Zhu, Isabel Najera, Fernando Garcia-Alcalde, Jianjun Niu

**Affiliations:** ^1^ Xiamen Center for Disease Control and Prevention, Xiamen, Fujian 361021, China; ^2^ School of Public Health, Fujian Medical University, Fuzhou, Fujian 351022, China; ^3^ Xiamen Hospital of Traditional Chinses Medicine, Xiamen, Fujian 361001, China; ^4^ Roche Pharma Research and Early Development, Immunology, Inflammation and Infectious Diseases, Roche Innovation Center Shanghai 201203, China; ^5^ Roche Pharma Research and Early Development, Immunology, Inflammation and Infectious Diseases, Roche Innovation Center Basel 4070, Switzerland; ^6^ Zhongshan Hospital, Xiamen University, Xiamen, Fujian 361004, China

**Keywords:** hepatitis B virus, mannose-binding lectin, mutation, quasispecies complexity, treatment naïve

## Abstract

Mannose binding lectin (MBL) plays important role in the innate immunity of human. Mutations in the MBL2 gene can significantly change the serum level of MBL, and consequently alter the susceptibility and progression of infectious disease. However, the association between the MBL2 profile and the HBV mutation and quasispecies complexity has not yet been reported. Our approach includes the study of the MBL2 gene genotype as well as ultra-deep sequencing of the HBV viruses obtained from the plasma of 50 treatment naïve patients with chronic HBV infection. We found that the liver function was better among patients within the high MBL2 group with respect to those within the medium/low MBL2 group. Likewise, the number of mutations in the HBV X gene as well as the viral quasispecies complexity were significantly higher in medium/low MBL2 production group. Nucleotide substitution rates were also higher within the medium/low MBL2 production group in all positions described to have an influence in liver cancer development, except for A1499G. In this work we show that the MBL2 profile may have an impact on the HBV X gene mutations as well as on viral quasispecies complexity.

## INTRODUCTION

Mannose binding lectin (MBL) in humans is capable of activating the complement system, triggering the immune response of MBL-associated serine protease (MASP). MBL can recognize and bind carbohydrate patterns which can be found on a large amount of pathogenic micro-organisms, including bacteria and viruses. The mutations of MBL2 gene, especially the structural variations in exon 1, can significantly reduce the level of functional MBL2 level in human serum by disrupting the collagenous structure of the protein [1]. Moreover, three polymorphisms in promoter region can also lead to the reduction of MBL2 concentration. The distribution of haplotypes constructed by these MBL2 polymorphisms vary among different races, therefore, the difference on MBL2 serum level can be observed between and within population [2]. The MBL2 deficiency caused by these polymorphisms can seriously compromise the innate immunity and also closely associated with susceptibility and resistance to infection by pathogens [3], including hepatitis B virus (HBV).

Mannose-rich oligosaccharides can be found on the surface of HBV proteins where MBL2 can bind and activate opsonization [4]. Epidemiological studies suggested that the −221 Y/X polymorphism is associated with chronic HBV infection, while the wild type carriers of promoter and exon 1 SNPs have significant higher natural recovery rate when comparing with the mutant carriers [5]. Similarly, evidence about the relationship between MBL2 polymorphism and HBV infection also has been obtained when comparing patients with chronic HBV infection and healthy individuals. It was shown that the case group has higher frequency of MBL2 haplotypes with low MBL2 production [6], therefore it can be assumed that MBL2 has a protective effect against HBV infection by direct clearance and inhibition of inflammatory liver damage. However, so far there is no certain conclusion on the association between HBV infection susceptibility and MBL2 polymorphisms. A recent meta-analysis reported null association between exon 1 polymorphism and chronicity, as opposed to the association found between the MBL2 genotype and progression of HBV infection, including liver cirrhosis and severe hepatitis B [7].

HBV genome is made of circular DNA, and its replication process is involved with pregenomic RNA and a specialized reverse transcriptase. Due to the absence of proofreading function, the HBV exhibits higher mutation rate when comparing with other DNA viruses [8]. This very specific property gives the possibility of co-existence within the same host of a group of HBV strains closely related, also known as quasispecies. The dynamic of HBV quasispecies is significantly affected by anti-viral treatment and host immune pressure, and also closely related with the progression of HBV infection. It has been confirmed that the patients with liver cirrhosis have higher quasispecies variability than chronic HBV infectors [9]. Moreover, quasispecies complexity can be affected by the dose and type of anti-viral treatment, and vice versa the efficacy of anti-viral treatment can be altered by the complexity. In order to gain a better sight of the association between the polymorphisms in MBL2 and the mutation status and quasispecies of HBV, and to eliminate the possible confounding effect caused by anti-viral treatment, we conducted a study among 50 treatment naïve patients with chronic HBV infection by using MBL2 genotyping assay and HBV ultra-deep sequencing.

## RESULTS

### Construction of MBL2 haplotypes

Phase 2.1 employs Bayesian algorithm to estimate the haplotype for each patient, and the results showed that among 50 subjects, 22 (44%) were assigned to high MBL2 group and 28 (56%) were classified as medium/low MBL2 group in accordance with the classification criteria reported in method section. Detailed information about haplotypes for enrolled subjects was demonstrated in Table 1.

**Table 1:** The MBL2 haplotype distribution among treatment naive patients with chronic HBV infection

MBL2 group	Haplotypes	Number of Patients N(%)	Total
High MBL2 group	HY/HY	8(16%)	
	HY/LY	14(28%)	22(44%)
Medium/Low MBL2	LY/LY	6(12%)	
Group	HY/LX	7(14%)	
	LY/LX	6(12%)	
	HX/HX	1(2%)	
	HY/HX	3(6%)	
	LX/LX	5(10%)	28(56%)

### Comparison on the characteristics of enrolled patients

Student's T test was performed to compare the difference of the mean age between two groups, and the mean age of high MBL2 group was 25.64 years and 28.93 years for medium/low MBL2 group, significance was detected. As for gender, no significance was found when comparing the distribution of two groups. The alanine aminotransferase (ALT) was found significantly higher in medium/low MBL2 group than high MBL2 group. Similar trend was also found in the comparison on the aspartate transaminase between two groups. The mean of plasma MBL2 level in high MBL2 group was 2373.27 ng/ml, while the corresponding level in medium/low MBL2 group was 1337.86 ng/ml. The results of Student's T test suggested that the difference was significant, indicating that the MBL2 level was significantly suppressed in medium/low MBL2 group when comparing with those subjects with high MBL2 haplotypes (*P*=0.002). Moreover, the statistical analysis also revealed that the X gene mutation was significantly frequent in medium/low MBL2 group than high MBL2 group (See Table 2 and Figure 1).

**Table 2:** The characteristics of treatment naive patients with chronic HBV infection

Variable	High MBL2 group	Medium/Low MBL2 group	*P* Value
Age^#^	25.64±4.327	28.93±6.164	0.039*
Gender			
Male	11	17	
Female	11	11	0.449
ALT(U/L)^#^	41.14±28.97	72.81±53.25	0.015*
AST(U/L)^#^	30.51±13.66	52.93±37.14	0.010*
MBL2 level(ng/ml)^#^	2373.27±772.88	1337.86±1315.03	0.002*
X gene mutation^#^	20.68±11.28	38.68±18.20	0.000*

* *P*<0.05

# (Mean±SD)

**Figure 1:**
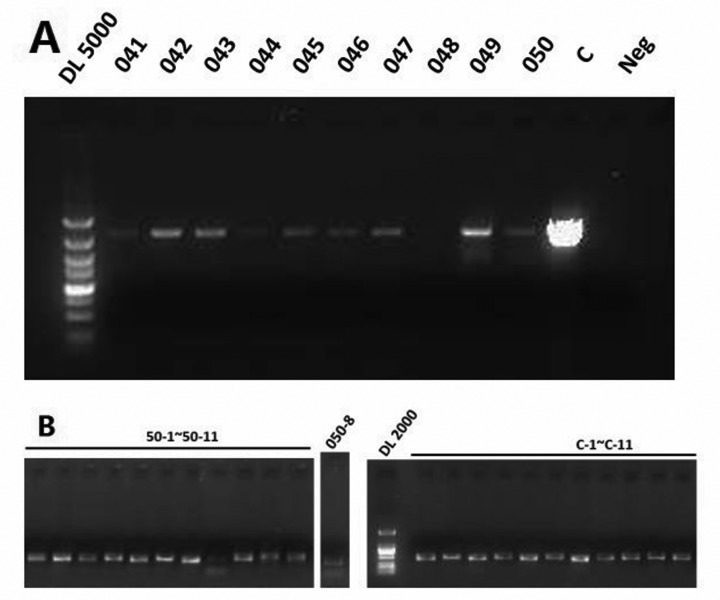
(**A**) The first round PCR product of subjects 41-50. (**B**) The 11 overlapping fragments of subject 50.

### Comparison on the X gene mutation rate

**Figure 2:**
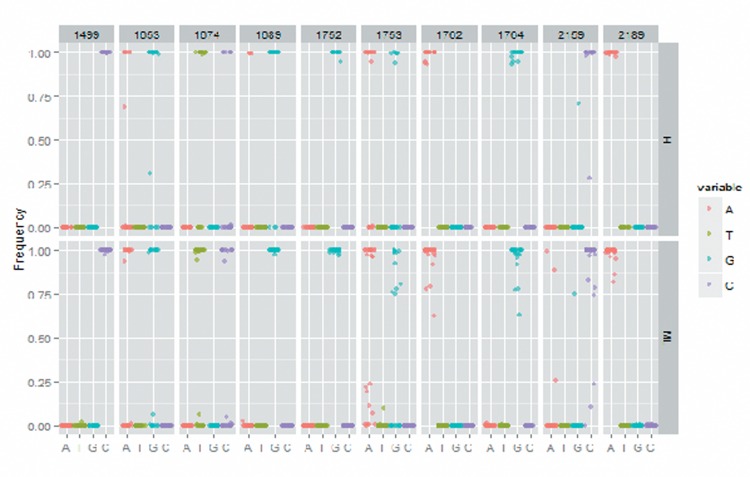
The X gene mutation rate in high MBL2 group and medium/low MBL2 group.

No significant difference was found in the mutation rate of T1689 and C1752 between two groups. As for A1499, the statistical analysis suggested that the mutation rate was significantly higher in high MBL2 group than medium/low MBL2 group. However, we found that the medium/low MBL2 group has higher mutation rate in T1653, T1674, T1753, A1726T and G1764A when being compared with high MBL2 group, and significance was observed (Table 3 and Figure 2).

**Table 3:** The comparison of X gene mutation rate between groups divided by MBL2 haplotypes

Nucleotide	High MBL2 group (Mean Rank)	Medium/low MBL2 group (Mean Rank)	*P* Value
*A1499G*	31.68	20.64	0.008*
*T1653*	20.50	29.43	0.002*
*T1674*	20.07	29.77	0.007*
T1689	26.55	24.68	0.587
C1752	25.52	25.48	0.992
T1753	20.52	29.41	0.023*
A1762T	19.11	30.52	0.004*
G1764A	19.07	30.55	0.004*

* *P*<0.05

### Comparison on quasispecies complexity of whole genome and X gene

**Figure 3:**
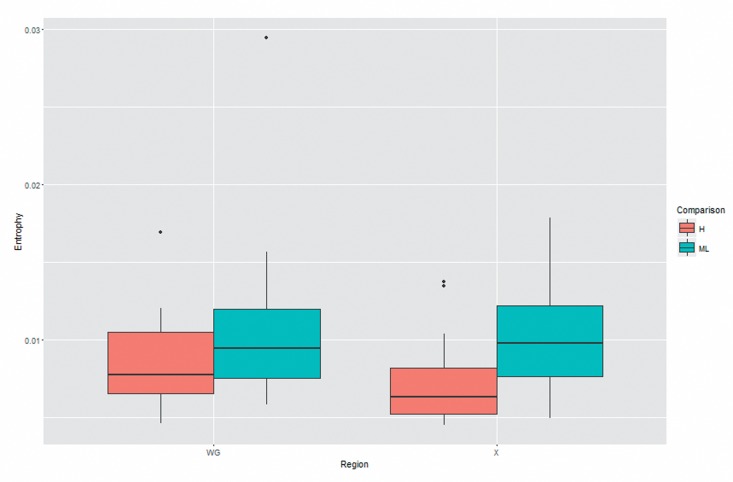
The comparison of quasispecies complexity between two groups.

Shannon entropy of whole genome and X gene was calculated by using the equation reported in method section. As demonstrated in Table 4 and Figure 3, we found both Shannon entropy of whole genome and X gene was significantly higher in medium/low MBL2 group than high MBL2 group, reflecting the HBV quasispecies complexity was higher in the patients with lower MBL2 production.

**Table 4:** The comparison of quasispecies complexity between groups divided by MBL2 haplotypes

Region	High MBL2 group	Medium/low MBL2 group	*P* Value
X gene	0.007±0.003	0.010±0.004	0.002*
Whole Genome	0.008±0.003	0.010±0.005	0.089

* *P*<0.05

## DISCUSSION

Our study investigated the relationship between human MBL2 haplotypes and the HBV X gene mutation profile, the nucleotide substitution rate of X gene, and viral quasispecies complexity. X gene can be qualified as the smallest open reading frame in whole HBV genome, its DNA sequence is highly conserved between subgenotypes of HBV. Therefore, it can be assumed that the HBx protein has important influence on the replication and viral life cycle. In addition, truncated X gene caused by HBV integration into human DNA may induce the occurrence and development of hepatocellular carcinoma [10]. The HBx protein can also induce supernumerary centrosomes and multipolar spindles, and consequently impair the centrosome integrity by inhibiting Crm1 which can increase the risk of hepatocellular carcinoma [11]. In a cell assay, HBx was found to be interacting with P53 directly, and leads to the inhibition of P53 transactivation [12], suggesting that HBx can impair the function of tumor suppressors and consequently elevate the hepatocellular carcinoma (HCC) risk. Given the critical importance of X gene in the carcinogenesis in HCC, we further compared the nucleotide substitution rate of some specified positions in X gene to investigate the relationship between MBL2 production and HCC related mutation.

The results revealed that patients with medium/low MBL2 haplotypes present a higher number of mutations in X gene and greater quasispecies complexity. As for the nucleotide substitution rate, medium/low MBL2 group has higher substitution rate in all investigated positions, except for mutation A1499G where the substitution rate was higher in high MBL2 production group.

In an epidemiological study conducted in Hong Kong, A1499G mutation has been associated with the risk of HCC, the data revealed that the mutation rate of A1499 was 20% in chronic HBV patients while it elevated to 62.5% among HCC cases [13]. But interestingly, among all 50 treatment naïve patients we enrolled the mutation rate of A1499 was significantly higher than 62.5% regardless of the MBL2 haplotype. Although the sample size is relatively small, it still provided some solid evidence for the investigation of HBV-related HCC risk among the population in an epidemic area of both HBV and HCC. The rest of nucleotide positions in X gene we investigated exhibited higher mutation rate in medium/low MBL2 group than high MBL2 group. The association between T1653 and the risk of HCC has been well documented, a case-control study reported a positive correlation among T1653 and HCC among Japanese population regardless the HBeAg status [14]. Its underlying mechanism may be related with the position in the HBV genome. Mutations occurred in that area can significantly increase the activity of core promoter and enhancer II [15], and consequently promote the replication of HBV. However, this mutation seems to have demographic associations: the investigation among 61 chronic HBV cases in Turkish population only found 0ne case with mutation, and in our study we observed 10 in 50. This mutation may also be attributed to the elevated HCC risk among population in Xiamen. The 1762 and 1764 double mutation has been identified as the major risk factor of HBV-related HCC, and the carcinogenesis mechanism involves with the down-regulation of HBeAg synthesis, up-regulation of pre-genomic RNA and the enhancement of HBV replication [16]. A case-control study which compared the double mutation between HCC cases and HBV-related liver disease patients found that the frequency of double mutation was elevated in HCC cases [17], in addition, prospective study with observation up to 13 years revealed that the double mutation at baseline was positively associated with the eventual development of cirrhosis or hepatocellular carcinoma, whereas the subjects without double mutation maintained better liver function at the end of follow up[18]. Aside from the carcinogenesis of the double mutation itself, double mutation also possesses synergistic effect with other mutations, a study conducted in Korea illustrated that the co-occurrence of T1653 and double mutation would significantly elevate the HCC risk when comparing with either T1653 or double mutation alone [19]. Our study indicated that high MBL2 serum level is capable of inhibiting the occurrence of double mutation, and alone with other mutations related with HCC, therefore, it can be assumed that high MBL2 may maintain protective effect against HBV-related HCC.

Consistent with the findings obtained from the comparison of nucleotide substitution, we found that the Shannon entropy of the HBV genomes was 0.008 in high MBL2 group and 0.010 in medium/low MBL2 group. Similarly, the Shannon entropy was significantly higher in subjects with medium/low MBL2 production. It has been reported that the quasispecies complexity of X gene is closely associated with the transactivation, and by comparing the HBV DNA sequence obtained from liver failure cases and chronic carriers, significant difference was found in the quasispecies complexity of X gene [20]. In general, the quasispecies can be observed in whole genome of HBV, and it is understood that higher HBV complexities present worse clinical responses. Moreover, the quasispecies also have impact on the anti-viral treatment, and may also lead to drug resistance by selection pressure caused by treatment. Our study revealed that the quasispecies complexity is inversely associated with MBL2 production, therefore, MBL2 profile may be a prognostic indicator for the HBV infection, as well as a potential therapy in future.

In conclusion, we found that MBL2 profile can significantly alter the mutation number and nucleotide substitution rate of X gene, as well as quasispecies complexity among the treatment naïve patients with chronic HBV infection. Due to the property of treatment naïve patients, the confounding effect caused by anti-viral treatment has been completely ruled out. Therefore, we can assume that the MBL2 has protective effect against the progression of HBV infection, and the MBL2 profile may alter the outcome of the disease. However, further studies are needed to clarify the association between MBL2 profile and X gene mutations.

## METHODS

### Study participants

50 treatment naïve patients with chronic HBV infection have been recruited between Apr 2013 and Oct 2013 from tertiary medical centers of Xiamen city. The HBV infection of patients was confirmed by using commercial enzyme-linked immunosorbent assay kits (Wantai BioPharm, Beijing, China) and quantified by using real time fluorescence quantitative PCR. Patients were included in this study if aged from 20 to 79 years old, HBV DNA≥10^7^ copies/ml and no prior history of anti-viral treatment. The exclusion criteria were as follows: the co-infection of HCV/HIV, received anti-viral treatment and refusal. The HCV/HIV status and prior treatment history were determined by reviewing medical record under the authorization of enrolled patients. All patients provided written, informed consent, in agreement with the Helsinki declaration and the policy of the Ethics Committee of Xiamen Center for Disease Control and Prevention approving this study.

### Sample collection and DNA extraction

All patients provided a 5-ml blood sample on the day of inclusion. Blood samples were centrifuged at 4000rpm for 10 min to separate serum and blood cells and stored at −78°C prior to DNA extraction. Human genome DNA and HBV DNA were extracted from blood cells and serum respectively by using Magna Pure LC 2.0 system (Roche Applied Science, Mannheim, Germany).

### MBL2 genotyping assay

Matrix-Assisted Laser Desorption/ Ionization Time of Flight Mass Spectrometry was performed to determine the 2 polymorphisms in MBL2 gene, namely −550 H/L and −221 Y/X. All procedures have been performed strictly under the user's manual (Sequenom, San Diego CA, USA). A negative water control and reference DNA were employed as quality control measures during the genotyping assay. In addition, approximately 10% of the samples were randomly selected and repeated for genotyping as duplicated controls. As a result, the genotyping call rate was 100%.

### Determination of MBL2 serum level

Human MBL2 quantikine ELISA kit (R&D systems, Minneapolis, MN, USA) was used to determine the serum level of MBL2. Following is the detailed procedure for MBL2 determination: (1) Firstly pipette 50 μl of assay diluent to each well. (2) Then pipette 50 μl of standard, control or plasma sample to each well, cover with plate sealer and incubate at room temperature for 2 hours. (3) Aspirate each well and wash, repeating the process 3 times for a total of 4 washes. (4) Add 100 μL of Conjugate to each well. Cover with a new plate sealer, and incubate at room temperature for 2 hours on the shaker. (5) Aspirate and wash for 4 times. (6) Add 100 μL Substrate Solution to each well. Incubate at room temperature for 30 minutes on the benchtop, and keep from the exposure of light. (7) Add 100 μL of Stop Solution to each well. Read at 450 nm within 30 minutes. Set wavelength correction to 540 nm or 570 nm. (8) Using the software provided by manufacturer to draw a standard curve and read the MBL2 concentrations for each sample.

### HBV ultra-deep pyrosequencing

In order to analyze the HBV mutation and quasispecies by ultra-deep pyrosequencing (UDPS), we designed a two-rounds PCR to amplify the HBV DNA. In the first round PCR, the whole 3.2kb genome of HBV was amplified; subsequently, we 11 overlapping fragments were amplified in the second round PCR. First round PCR was performed with 4ul of HBV DNA in a 50μL reaction mixture containing 5μL of expand high fidelity PCR buffer, 10mM dNTPs (1.0μL), 1.0μL for each primer (forward and reverse) [21], 0.5μL of expand high fidelity Taq and 37.5μL of ddH2O. Amplification condition consisted of 94°C for 2 min followed by 30 cycles of denaturation of 30 sec at 94°C, annealing of 1 min at 60°C, and extension of 3 min at 72°C, with final 5 min extension at 72°C. The 50μL reaction mixture of second round PCR containing 1.3μL of first round PCR product, 5μL of expand high fidelity PCR buffer, 10mM dNTPs (1.0μL), 2μL of primer contained UDPS barcode, 0.5μL of expand high fidelity Taq and 40.2 μL of ddH2O. Amplification condition for second round PCR was as follows: 94°C for 2 min followed by 22 cycles of denaturation of 30 sec at 94°C, annealing of 30 sec at 55°C, and extension of 40 sec at 72°C, with final 5 min extension at 72°C. Both two round PCR products have been detected and photographed by using agarose gel electrophoresis (See Figure1). The second round PCR products were then purified with AMPure beads (Beckman Coulter Inc., Brea, CA, USA) and subjected to deep sequencing performed using the GS FLX system (Roche Applied Science, Bandford, CT, USA) under the guidance of the manufacturer's instructions.

### Data analysis

Phase software version 2.1 was employed to perform MBL2 haplotype construction, and patients with HY/HY and HY/LY were assigned to High MBL2 group while those with LY/LY, HY/LX, LY/LX, HX/HX, HY/HX and LX/LX were classified as Medium/Low MBL2 group.

Reads were aligned and error-corrected to their corresponding consensus sequences by using MOSAIK and rc454. Quality control and visualization of the coverage profiles were assessed with Qualimap2. Variant calling was performed using V-Phaser 2 and V-Profiler and Mann-Whitney U test was employed to compare the difference between two groups. The complexity of HBV quasispecies was estimated by following equation:

$$\text{S}=-\frac{\sum_{\text{i}}^{\text{n}}=f_{i}(\text{ln}f_{i})}{\text{N}},$$

where *f_i_* is the frequency of a sequence variant and N is the length of the sequence. Both whole genome and X gene were assessed. Student's T-test was performed to compare the difference between two groups. The frequencies of categorical variables were analyzed with Chi-squared test. A P-value smaller than 0.05 was considered as significant. Unless specified, all statistical analyses were performed by using IBM Statistics SPSS version 19.

### Ethic approval

All enrolled patients provided written, informed consent, in agreement with the Helsinki declaration and the policy of the Ethics Committee of Xiamen Center for Disease Control and Prevention approving this study.
